# Supplementary material for: MetaRibo-Seq measures translation in microbiomes
Source: Nat Commun. 2020 Jun 29;11:3268. doi: 10.1038/s41467-020-17081-z (PMC7324362; doi:10.1038/s41467-020-17081-z)
Supplement: Supplementary file 10 — Supplementary Data 7 [file 41467_2020_17081_MOESM10_ESM.zip › File2/Confidence_High_Taxonomy/34647_out.krona.html]

Javascript must be enabled to view this page.

members
magnitude
magnitudeUnassigned
count
unassigned
taxon
rank

34647\_out

8

2
8
superkingdom

8
phylum
1239

186801
class
8

186802
8
order

186803
4
family

4
genus
2569097


SRS057478\_contig\_number\_contig-100\_7506.7507SRS063040\_contig\_number\_contig-100\_3086.161655SRS143342\_contig\_number\_contig-100\_7043.103152SRS148159\_contig\_number\_13147
39488
species
4

4
family
186806

genus
4
1730

SRS015431\_contig\_number\_contig-100\_9083.263169SRS018427\_contig\_number\_contig-100\_6039.128118SRS022713\_contig\_number\_contig-100\_20876.20876SRS144183\_contig\_number\_25712
